# A Retrospective Study of Hospital Recidivism Among Patients with Alcohol Use Disorders Treated with Intramuscular Naltrexone

**DOI:** 10.7759/cureus.6287

**Published:** 2019-12-04

**Authors:** Eduardo D Espiridion

**Affiliations:** 1 Psychiatry, Reading Hospital-Tower Health, West Reading, USA

**Keywords:** hospital recidivism, alcohol use disorders, naltrexone

## Abstract

Introduction

Alcohol use disorder is a chronic, relapsing condition that is associated with compulsive alcohol use and loss of control of alcohol intake. It is a common problem in the hospital setting. It has also become a public health dilemma. This study seeks to analyze the benefit of long-acting naltrexone. This well-studied agent is indicated for alcohol use disorder.

Methods

This was a retrospective cohort study between July 1, 2016, and October 31, 2017, using Meditech's Pharmacy Admission Report (MPAR), which is the community hospital's network's electronic medical record (EMR) system as the data source "alcohol use disorders" covers a broad spectrum of sub-diagnoses. The patients were selected after they were admitted with a primary diagnosis of alcohol abuse dependence (APDRG v34code).

Results

The readmission rate in the study population (intramuscular naltrexone) was 2.86% and readmission in the control population (standard of care) was 25.70%. Patients diagnosed with alcohol abuse dependence are at a significantly decreased risk for readmission if treated with intramuscular naltrexone (odds ratio (OR) 8.5%; 95% confidence interval (CI) 0.0115, 0.6300; p=0.0159).

Conclusion

This study showed that treating patients admitted under the diagnosis of alcohol abuse dependence with intramuscular naltrexone may be an effective intervention in reducing hospital readmission. Additional studies are warranted to clarify and establish optimal treatment strategies.

## Introduction

Alcohol use disorder is a chronic relapsing condition that is optimally treated using a combination of pharmacological and psychosocial treatments. Different treatment approaches addressing withdrawal symptoms as well as reducing cravings and consumption are routinely used to address this malady. However, alcohol use disorders have increasingly become a public health dilemma. It continues to be associated with morbidity and mortality. This has been made evident by the number of hospitalizations and health care costs. A 2014 surveillance report completed by the National Institute on Alcohol Abuse and Alcoholism (NIAAA), stated the aggregate annual cost for all hospital stays with an alcohol-related diagnosis totaled 33.4 billion US dollars [1]. The NIAAA, which is based in the United States of America, leads the national effort to reduce alcohol-related problems. According to a 2011 national analysis performed by the Agency of Healthcare Research and Quality (AHRQ), alcohol-related disorders represented the fifth most common condition with the highest 30-day readmissions among Medicaid patients (aged 18-64), at 26.6 per 100 admissions [2]. It was second among uninsured patients in the same category, at 16 per 100 admissions.

Definitive treatment methods are yet to be established due to the complex and relapsing nature of the disease. The evolving interplay between neurobiological and psychosocial factors poses a significant challenge in providing structured long-term care. There is some research on the efficacy of inpatient treatment of alcohol use disorder [3]. Currently, patients hospitalized due to alcohol use disorder are treated with largely supportive methods targeted at detoxification and monitoring for withdrawal symptoms. This may stabilize the patient and provide immediate resolution but does not address the underlying mechanism of the disease. Although alcohol use disorder is certainly a chronic illness, improved treatment in the acute setting may serve as an impetus for a meaningful long term recovery.

This study seeks to analyze the benefit of intramuscular naltrexone in serving this very purpose. We looked at the injectable long-acting formulation of naltrexone, a well-studied first-line agent in alcohol use disorder [4]. Naltrexone primarily works through the blockade of the mu-opioid receptor [5]. It is a synthetic cogener of oxymorphone with no opioid agonist properties. It is indicated for both opioid and alcohol use disorders.

Rate of hospital readmission was chosen as the measured comparison between treatment with the intramuscular naltrexone versus standard of care. Recidivism is not only a reflection of treatment efficacy but has also served as an established quality benchmark for hospital care. Through this study, I hope the impact of long-acting naltrexone on lowering readmission rates will be predictive on a larger scale.

## Materials and methods

This was a retrospective cohort study in a community hospital serving a metropolitan population in Maryland, the United States of America, between July 1, 2016, and October 31, 2017. I used the Meditech Pharmacy Admissions Report (MPAR), which is the hospital network's electronic medical record (EMR) system as the data source. The database contains records from consultation with practitioners, prescription information, care referral, and hospital administrations.

"Alcohol use disorders" cover a broad spectrum of sub-diagnoses. I selected patients who were admitted with the primary diagnosis of alcohol abuse dependence (All Patients Refined Diagnosis Related Groups (APR DRG) v34 code = 775). This diagnosis is inclusive of both acute alcohol abuse as well as acute on chronic abuse/dependence. The diagnostic and statistical manual of mental disorders (DSM) fourth edition was used as well. 

I excluded patients with confounding diagnoses, such as opioid dependence or stimulant dependence, and those who received alternative pharmacologic treatment. A total of 393 patients remained that fit my criteria.

These patients were divided into two cohorts: a control population (n=358) whose treatment was supportive care and detoxification and a study population (n=35) who were treated during their hospital admission with 380 mg of intramuscular naltrexone. This is a random sampling of the intended population for the study. Patient encounters were then followed up for readmission, defined as rehospitalization within 30 days of discharge. The readmissions definition comes from the Health Service Cost Review Commission (HSCRC) and Chesapeake Regional Information System for our Patients (CRISP) in their RY 18-Potentially Avoidable Utilization logic. The RY 18-Potentially Avoidable Utilization logic includes information about hospital care that is unplanned and can be prevented through improved care, care coordination, or effective community-based care. HSCRC is an independent Maryland state agency that has a broad responsibility regarding the public disclosure of hospital data and operation performance. Additionally, I used CRISP to cross-reference patients to see if a readmission fell outside our metropolitan hospital network. CRISP is a regional health information exchange serving Maryland.

## Results

Patients were screened from the inpatient units of a community hospital. This included patients in the inpatient behavioral health unit as well as the inpatient medical units. Patients from the outpatient department were not included in the study. Alcohol abuse dependence as defined by the APR DRG coding system was the primary diagnosis of the patients included in the study. The injectable naltrexone patient population included patients who received 380 mg intramuscular naltrexone during their inpatient stay (n=35). They were identified from the discharged inpatient encounters from July 1, 2016, to October 31, 2017. The control group included the discharged inpatient encounters from July 1, 2016, to October 31, 2017, and patients were primarily treated with alcohol detoxification without the intramuscular naltrexone injection (n=358). The exclusion criteria included patients with significant comorbidities, which could act as confounding factors, including other substance use disorders. Patients treated with other pharmacological modalities were excluded. This included patients treated with acamprosate (n=10) and disulfiram (n=2).

The diagnosis of alcohol abuse dependence was established in 393 patients. Of those, 35 were treated using 380 mg intramuscular naltrexone injection while the remaining 358 were treated with supportive therapy and detoxification. The review of the patient data did not disclose any information as to the number of patients from each group who eventually transferred to inpatient alcohol rehabilitation programs. Also, none of the patients went home against medical advice.

The readmission rate in the study population (intramuscular naltrexone treatment) was 2.86%, and readmission in the control population (standard of care) was 25.70%. The group treated with intramuscular naltrexone is at a statistically significant decreased risk for readmission (OR 8.5%;95% CI 0.0115, 0.6300; p=.0159). This study used the rates of hospital recidivism as a measure of the efficacy of the intramuscular naltrexone by comparing it to patients diagnosed with alcohol use disorders treated with supportive care and detoxification.

## Discussion

This study showed that treating patients admitted under the diagnosis of alcohol abuse dependence with intramuscular naltrexone may be an effective intervention in reducing hospital readmission. Long-acting naltrexone was well-tolerated and resulted in a reduction in heavy alcohol drinking among treatment-seeking alcohol-dependent patients during six months of treatment [4].

Those treated with the standard of care were readmitted at a rate of 25.7%, as opposed to the 2.86% readmitted after the naltrexone injection. This substantial discrepancy warrants further analysis into defining the exact role of naltrexone injection in care for the growing patient population suffering from alcohol use disorders.

There were several limitations that should be addressed should a larger scale study take place. One such limitation is that my readmission criterion was "all-cause readmission." This is because, when using CRISP for readmission data, I could not access the cause of the admission for de-identified patients. Therefore, I chose to use "all-cause readmission" for all patients included in the study. A second limitation is the population was not controlled for age and gender. Given that the study population was low (n=35), I could not account for these variables. Finally, I did not account for when the naltrexone injection was administered in the study population during their hospital stay.

Alcohol dependence remains to be a major public health problem. It remains one of the leading causes of disability worldwide [6]. It significantly contributes to the disease burden as a result. Pharmacologic treatment options are few. The management of this condition involves a combination of pharmacologic therapy and psychosocial support. Alcoholism is a leading cause of disability worldwide. In one study, acamprosate has shown to significantly reduce the risk of any alcohol drinking [7]. It should be started once abstinence is achieved, as it ameliorates some symptoms associated with abstinence and withdrawal, and, therefore, patients may notice less of a desire to return to drinking in that circumstance. There are issues with treatment compliance, as the medication has to be administered three times a day. Clinical trials with disulfiram have found lower rates of relapse to drinking in those who are compliant with the medication [8]. Disulfiram biologically leads to adverse effects when combined with alcohol intake and should only be used by abstinent patients with the goal of maintaining abstinence. Both disulfiram and acamprosate are not compatible with alcohol drinking. Naltrexone can be initiated even while the individual is still drinking alcohol, and it is the only one available as a long-term injection among the available pharmacologic treatment options. This allows for the treatment to be initiated at the point of maximum crisis without the need for enforced abstinence or medically supervised withdrawal. In another study, naltrexone long-acting formulations provided a new opportunity to improve the efficacy, delivery, and safety of treatment to alcohol-dependent patients by enhancing medication compliance, diminishing adverse events, and reducing fluctuating plasma naltrexone levels [9].

Naltrexone is a competitive, non-selective opioid antagonist that blocks the reinforcing effects of opioids. This endogenous opioid system modulates the transmission of dopaminergic neurons. The neurotransmitter dopamine contributes to feelings of pleasure and satisfaction as part of the reward system. This, in turn, results in a positive reinforcement that plays a part in substance liking and seeking behaviors. The endogenous opioids influence the motivational and mood-regulating systems as a result [10]. The reinforcing effects of alcohol associated with its abuse liability are mediated by the dopaminergic pathways that originate in the ventral tegmental area, relay to the nucleus accumbens with neuronal inputs from other limbic regions, and progress to the cortex [11]. Naltrexone decreases alcohol reinforcement by the suppression of alcohol-mediated beta-endorphin stimulation of dopamine neurons directly in the nucleus accumbens and reduction of the beta-endorphin disinhibition of the tonic inhibition of dopamine cells by gamma-aminobutyric acid neurons in the ventral tegmental area [12]. It also found that short-term treatment of naltrexone decreases the chance of alcohol relapse by 36% and is likely to reduce the chance to return to drinking for 13% [12]. Strategies to improve adherence should be concomitantly given. This includes the psychosocial interventions and management of side effects. Patients who experience multiple relapses despite high-intensity continuing care may benefit from extended-release naltrexone. An open-label randomized controlled trial examined the effectiveness of extended-release naltrexone versus oral naltrexone for alcohol dependence treatment in primary care [13]. Their conclusion suggests that extended-release naltrexone was potentially more efficacious, feasible, and cost-effective than oral naltrexone when treating community-dwelling persons with alcohol use disorders. The use of long=acting injection formulation contributed to treatment compliance particularly with patients who are unable to achieve abstinence due to a failure to adhere to a daily medication regimen. In another study, it showed that the extended-release naltrexone, in combination with psychosocial intervention, was associated with improvements in the quality of life, specifically in the domains of mental health, social functioning, general health, and physical functioning [14]. It is not yet known how long alcohol-dependent patients who respond to naltrexone treatment should continue their treatment. For patients who do not achieve remission or a significant reduction in heavy drinking, switching medications is recommended. At the moment, there are still few choices to medically manage alcohol use disorder. 

## Conclusions

This study showed that treating patients admitted under the diagnosis of alcohol abuse dependence with intramuscular naltrexone may be an effective intervention in reducing hospital readmission. Those treated with the standard of care were readmitted at a rate of 25.7%, as opposed to the 2.86% readmitted after the intramuscular naltrexone injection. This substantial discrepancy warrants further analysis into defining the exact role of intramuscular naltrexone in care for the growing patient population suffering from alcohol use disorders. Additional studies should control for age and gender, as well as control for reason of admission.
